# Acute colonic pseudo-obstruction with transient failure of oral desmopressin in adipsic arginine vasopressin deficiency

**DOI:** 10.1210/jcemcr/luag334

**Published:** 2026-10-27

**Authors:** Wenyuan Li, Shahamah Ahmed, Sana Saeed, Mohammad Aziz

**Affiliations:** Department of Internal Medicine, Montefiore Medical Center, Bronx, NY 10466, USA; Department of Internal Medicine, Montefiore Medical Center, Bronx, NY 10466, USA; Department of Endocrinology, Montefiore Medical Center, Bronx, NY 10461, USA; Department of Endocrinology, Montefiore Medical Center, Bronx, NY 10461, USA

**Keywords:** adipsic arginine vasopressin deficiency, desmopressin, acute colonic pseudo-obstruction, Ogilvie syndrome, hyponatremia

## Abstract

We report the case of a middle-aged male with panhypopituitarism and adipsic arginine vasopressin deficiency (AVP-D) who presented with severe hyponatremia. During the correction of hyponatremia, he developed acute colonic pseudo-obstruction (ACPO or Ogilvie syndrome). Management included a bowel regimen, rectal tube placement, and endoscopic decompression. During hospitalization, repeated transitions from intravenous to oral desmopressin were consistently followed by brisk hypotonic polyuria and hypernatremia, raising concern for impaired enteral desmopressin absorption. After gastrointestinal recovery and separation of oral desmopressin and polyethylene glycol (PEG) by more than 4 hours, oral desmopressin again produced sustained antidiuresis. This case suggests that ACPO may transiently impair oral desmopressin absorption and that concurrent PEG administration may further reduce its efficacy.

## Introduction

Acute colonic pseudo-obstruction (ACPO), or Ogilvie syndrome, is characterized by marked colonic dilation in the absence of mechanical obstruction [1, 2]. Although its pathophysiology is incompletely understood, ACPO is thought to result from autonomic dysregulation leading to impaired colonic motility [2]. It most commonly occurs in hospitalized patients with severe medical illness, recent surgery, or metabolic disturbances [3, 4] and may progress to bowel ischemia or perforation if not promptly recognized and treated.

Patients with adipsic arginine vasopressin deficiency (AVP-D) are particularly vulnerable to disturbances in water homeostasis because they rely on prescribed fluid intake and exogenous desmopressin to maintain plasma osmolality [5, 6]. Consequently, factors that impair desmopressin absorption or effectiveness may produce clinically significant fluctuations in serum sodium.

We report a patient with panhypopituitarism and adipsic AVP-D who developed ACPO during correction of profound hyponatremia, followed by recurrent hypotonic polyuria and hypernatremia during transitions from intravenous to oral desmopressin. We discuss acute gastrointestinal dysmotility as a potential cause of impaired oral desmopressin absorption and explore whether polyethylene glycol (PEG 3350) may have further reduced absorption.

## Case presentation

A 46-year-old man with a history of adult-onset adamantinomatous craniopharyngioma (WHO grade I; 5.3 × 3.3 × 3.9 cm), for which he underwent transsphenoidal resections in 2014 and 2022, and external beam radiation therapy in 2024 with treatment course complicated by obstructive hydrocephalus requiring a ventriculoperitoneal (VP) shunt, blindness, panhypopituitarism, and adipsic AVP-D, presented with 1 week of progressive confusion, 3 days of emesis, and diarrhea. The patient had multiple hospitalizations for hyponatremia since 2024, most recently 2 weeks before the current admission. Following that admission, he was discharged on oral desmopressin 0.3 mg twice daily and prescribed free-water intake of 1800 mL/day. However, according to his spouse, he received approximately 2000 mL/day for 1 week after discharge. When a postdischarge laboratory test demonstrated mild hyponatremia of 134 mEq/L (SI: 134 mmol/L) (reference range, 135-145 mEq/L [SI: 135-145 mmol/L]), his prescribed free-water intake was reduced to 1500 mL/day. Three days before the current admission, he developed emesis and diarrhea, making adherence to the prescribed free-water intake difficult to assess, although his spouse stated that he was adhering to the 1500 mL/day restriction.

On physical examination, he was confused and hypothermic to 92.7 °F (SI: 33.7 °C). Initial laboratory tests showed profound hyponatremia of 106 mEq/L (SI: 106 mmol/L), with serum osmolality of 226 mOsm/kg (SI: 226 mmol/kg) (reference range, 275-295 mOsm/kg [SI: 275-295 mmol/kg]) (Fig. 1) and urine osmolality of 480 mOsm/kg (SI: 480 mmol/kg) (reference range, 300-1300 mOsm/kg [SI: 300-1300 mmol/kg]).

**Figure 1:**
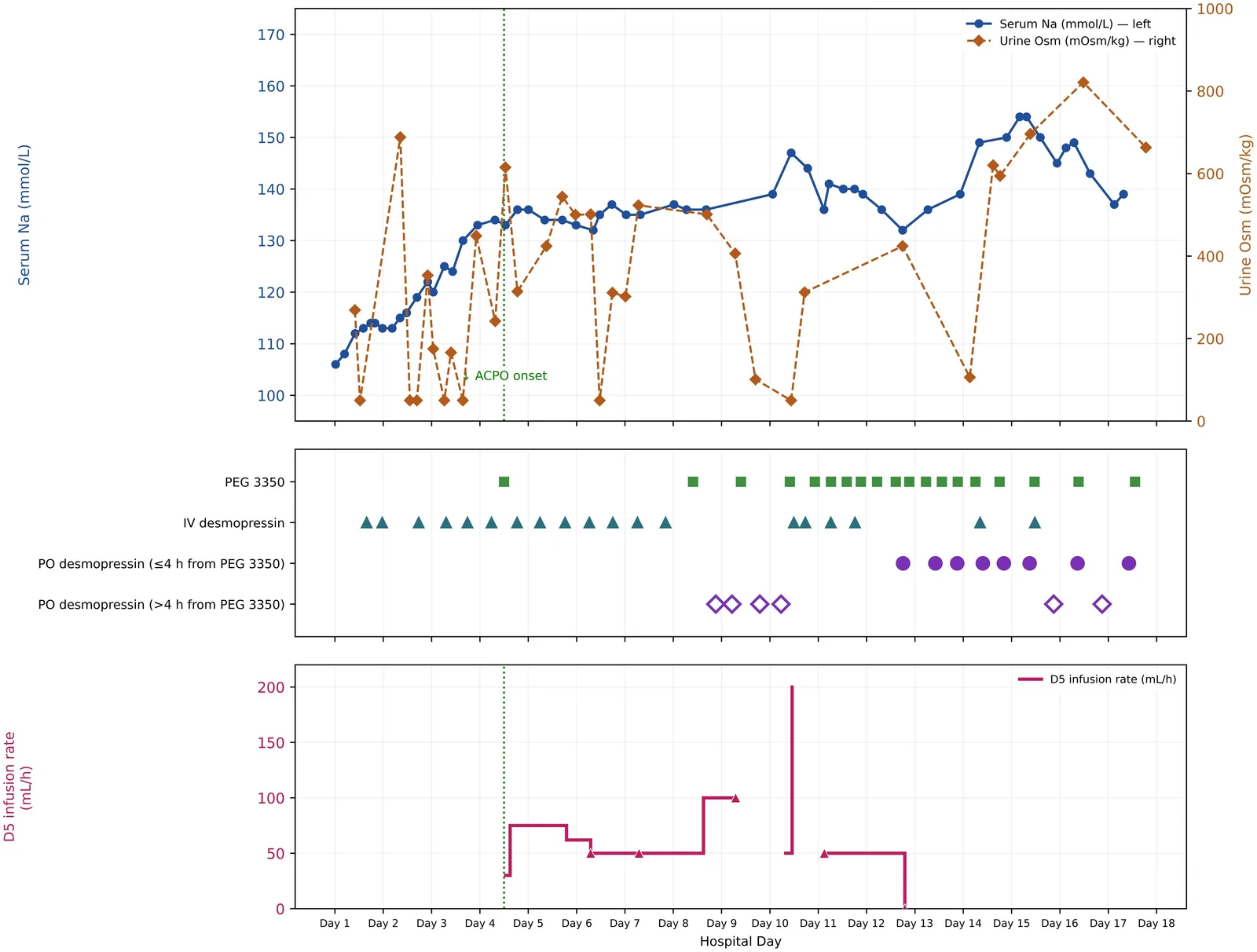
Serum sodium, urine osmolality, desmopressin and polyethylene glycol 3350 administration, and D5 infusion rate during hospitalization. During the first 4 days of hospitalization, hyponatremia was corrected with fluid restriction and intermittent intravenous desmopressin. Following the onset of ACPO, the patient was kept NPO and treated with intravenous desmopressin, rectal tube placement, and endoscopic decompression. Oral desmopressin doses are categorized according to whether they were administered ≤4 hours or >4 hours from the nearest PEG 3350 dose. The D5 infusion rate was adjusted during periods of NPO status or poor oral intake. ACPO, acute colonic pseudo-obstruction; D5, 5% dextrose; IV, intravenous; NPO, nil per os; PEG 3350, polyethylene glycol 3350; PO, oral.

## Diagnostic assessment

The patient was admitted to the intensive care unit (ICU) for severe hyponatremia. Computed tomography (CT) of the head showed no acute intracranial pathology. A stable 1.6-cm suprasellar lesion compatible with residual craniopharyngioma was present, and the VP shunt catheter was unchanged in position. Initial abdominal radiographs (Fig. 2A and 2B) and CT of the abdomen and pelvis showed no evidence of bowel obstruction or colitis. Cortisol and free thyroxine levels were within their respective reference ranges. His diarrhea resolved shortly after ICU admission.

**Figure 2:**
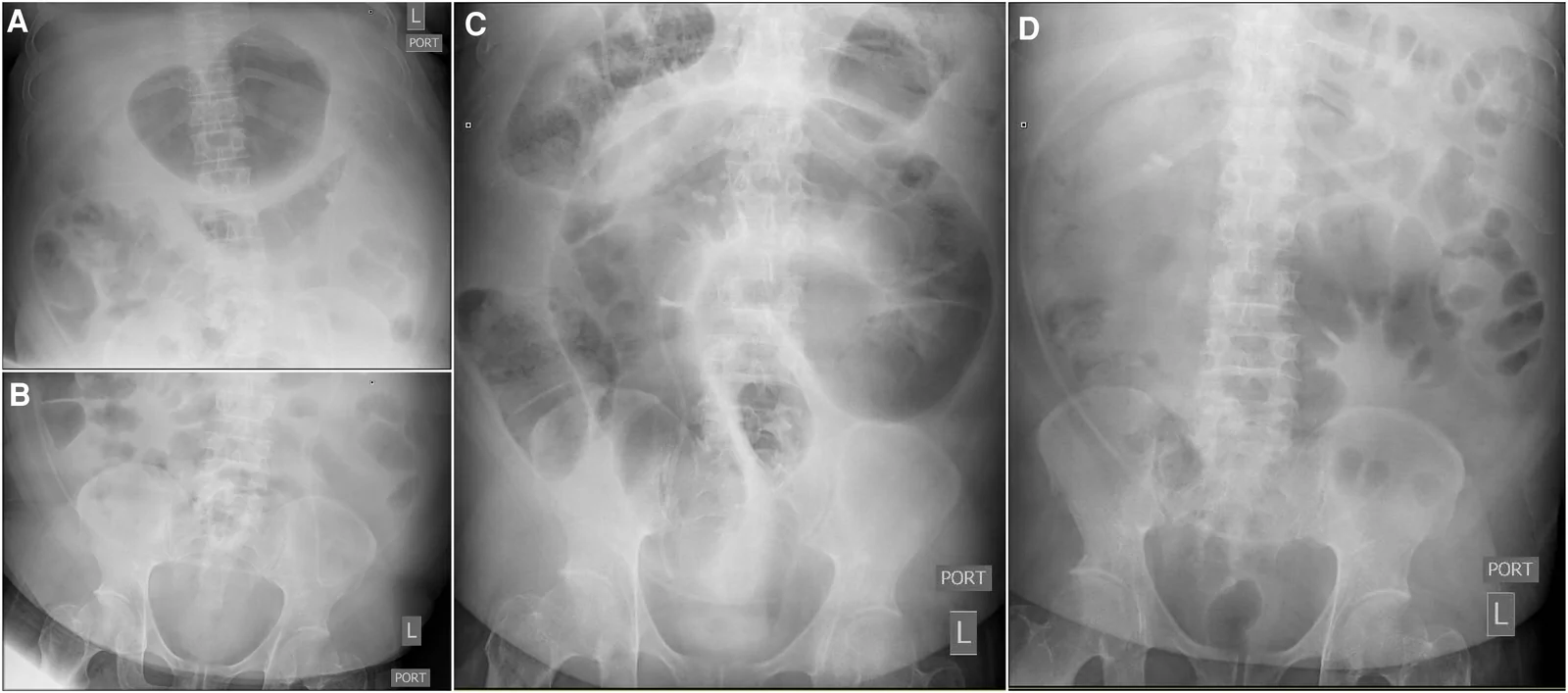
Abdominal radiographs during the development and treatment of ACPO. (A, B) Initial abdominal radiographs obtained on admission showing no evidence of bowel obstruction. (C) Abdominal radiograph obtained after the onset of ACPO, demonstrating diffuse colonic dilation. (D) Follow-up abdominal radiograph demonstrating improvement in colonic dilation after treatment. ACPO, acute colonic pseudo-obstruction.

## Treatment

Initial management focused on cautious sodium correction, targeting an increase of 6-8 mEq/L (SI: 6-8 mmol/L) per 24 hours, using fluid restriction and intermittent desmopressin dosing to prevent overcorrection of sodium. During the first 3 days, total daily fluid intake was limited to approximately 250 mL of intravenous fluid, while urine output was approximately 2300 mL/day. After improvement in mental status and correction of serum sodium to 124 mEq/L (SI: 124 mmol/L), the patient was transferred to the medical ward for further management, where progressive abdominal distension was noted. Abdominal radiography (Fig. 2C) demonstrated diffuse colonic dilation consistent with ACPO without evidence of mechanical obstruction. He was maintained nil per os (NPO) with Malecot rectal tube placement followed by endoscopic decompression. Neostigmine was withheld because of asymptomatic bradycardia (49 beats/minutes). To maintain free-water balance, he received a continuous intravenous 5% dextrose (D5) infusion while he remained NPO and when his oral water intake was poor.

## Outcome and follow-up

The patient's colonic dilation improved (Fig. 2D) 10 days after ACPO onset. By hospital Day 4, his serum sodium had normalized. During hospitalization, each attempt to transition from intravenous to oral desmopressin was followed by brisk hypotonic polyuria and hypernatremia, with prompt resolution of hypotonic polyuria after intravenous desmopressin was resumed (Fig. 1). Impaired gastrointestinal absorption was suspected to contribute to the difficulty in transitioning to oral desmopressin, with temporally proximate PEG 3350 administration considered an additional potential contributor during the second transition.

The patient's adipsia also complicated maintenance of normal serum sodium concentration and weaning of the D5 infusion. Scheduled water administration resulted in more consistent water intake and facilitated discontinuation of D5 infusion. At discharge, a stable serum sodium concentration had been achieved with a strict 1.5-L/day water prescription and oral desmopressin 0.3 mg twice daily.

## Discussion

Water homeostasis is maintained through 2 osmoregulatory mechanisms: AVP secretion and thirst [7]. Small increases in plasma osmolality are sensed by osmoreceptors located in the organum vasculosum of the lamina terminalis [8] and adjacent anterior hypothalamic structures, triggering both AVP release and thirst at similar osmotic threshold of approximately 280-290 mOsm/kg (SI: 280-290 mmol/kg) [9, 10]. AVP promotes renal water conservation, whereas thirst drives voluntary water intake to restore plasma osmolality. In adipsic AVP-D, hypothalamic injury—in this case, prior transsphenoidal surgeries and pituitary radiation—disrupts both AVP secretion [11] and the thirst response [9]. Consequently, water balance depends entirely on prescribed fluid intake and exogenous desmopressin rather than physiologic feedback. This unique physiology makes patients particularly vulnerable to significant fluctuations in serum sodium when either fluid intake or desmopressin effectiveness changes.

ACPO is an acquired disorder of colonic dysmotility that typically occurs in hospitalized patients with serious medical or surgical illnesses. Established risk factors include recent surgery, severe infection, neurologic disease, immobilization, opioid or anticholinergic medications, and metabolic disturbances, particularly hypokalemia, hypocalcemia, and hypomagnesemia [3, 4]. Although profound hyponatremia is less commonly recognized as a precipitating factor, several reports have suggested that severe hyponatremia and its correction may contribute to colonic dysmotility through alterations in neuromuscular excitability and autonomic regulation [3, 4]. In our patient, the temporal relationship among profound hyponatremia, gradual sodium correction, and subsequent development of ACPO suggests that the sodium disturbance and its correction may have contributed.

Management of ACPO follows a stepwise approach [12]. Conservative measures include bowel rest and nasogastric or rectal decompression, together with correction of underlying electrolyte and metabolic abnormalities. Neostigmine is the first-line pharmacologic therapy for cases that do not respond to conservative measures, with concurrent cardiac monitoring because of the risk of bradycardia [3, 4]. This patient was treated with bowel rest, rectal tube placement, and successful endoscopic decompression. Neostigmine was withheld because of baseline asymptomatic bradycardia.

The most distinctive feature of this case was the reproducible loss of oral desmopressin efficacy during acute gastrointestinal dysmotility. Each transition from intravenous to oral desmopressin was consistently followed by brisk hypotonic polyuria and rising serum sodium concentrations, whereas intravenous desmopressin promptly restored antidiuresis. Oral desmopressin has low bioavailability, estimated at only 0.08-0.16% relative to intravenous administration [13], making its clinical effect highly dependent on adequate gastrointestinal absorption. Previous pharmacokinetic studies have demonstrated that gastrointestinal transit significantly influences desmopressin absorption: slowing intestinal transit with loperamide substantially increases systemic exposure, whereas even physiologic delays associated with food intake reduce both the rate and extent of absorption [14, 15]. Although direct evidence in ACPO is lacking, severe gastrointestinal dysmotility and recent endoscopic manipulation may further impair the already limited absorption of oral desmopressin. The reproducible association between oral dosing and recurrent hypotonic polyuria in our patient supports the possibility that impaired gastrointestinal absorption, rather than a change in desmopressin requirement, was responsible for the observed loss of efficacy.

Polyethylene glycol (PEG 3350), administered after endoscopic decompression to reduce the risk of recurrent ACPO, may have been an additional contributing factor. By accelerating gastrointestinal transit and increasing luminal fluid volume, PEG-based preparations may reduce absorption of coadministered oral drugs [16-21]. No prior study has specifically examined PEG 3350 and oral desmopressin. In our patient, review of the medication administration record identified 2 instances in which transition to oral desmopressin was followed by brisk hypotonic polyuria. During the first failed transition to oral desmopressin (Days 9 and 10; Fig. 1), oral desmopressin and PEG 3350 were separated by more than 4 hours, suggesting that intrinsic gastrointestinal dysmotility may have impaired absorption. Incomplete ingestion of the medication was also possible because the patient had a degree of intellectual disability, compounded by frustration related to hospitalization and frequent blood draws, and may have pocketed or spit out medications. During the second transition (Days 13 and 14; Fig. 1), multiple PEG 3350 doses were administered within 4 hours of oral desmopressin, followed by rising serum sodium and a parallel change in urine osmolality. Concurrent weaning of D5 may also have contributed. After the medications were separated by more than 4 hours, oral desmopressin again produced consistent antidiuresis. Although this temporal association does not establish causality, it is consistent with the recognized effect of PEG 3350 on oral drug absorption and, to our knowledge, represents the first clinical description of a possible interaction with desmopressin in the setting of active ACPO.

Several strategies may help reduce the risk of ACPO in patients undergoing correction of severe hyponatremia: gradual sodium correction with frequent electrolyte monitoring; early correction of hypokalemia, hypocalcemia, and hypomagnesemia, which are independently associated with colonic dysmotility; encouragement of early mobilization; and maintaining a low threshold for serial abdominal examination and imaging in patients with adipsic AVP-D undergoing rapid electrolyte shifts. For patients outside the inpatient setting who require oral desmopressin during periods of active or anticipated gastrointestinal dysmotility, intranasal desmopressin may be a reasonable alternative because it bypasses the gastrointestinal tract and has higher bioavailability than the oral formulation (approximately 3-5% vs 0.08-0.16%) [13]. However, nasal congestion, mucosal edema, or prior sinonasal surgery may limit its reliability. Intravenous or subcutaneous administration remains preferred when feasible if gastrointestinal absorption is a concern.

This case illustrates 2 clinically important observations. First, severe hyponatremia and its correction may contribute to the development of ACPO in susceptible patients. Second, clinicians should be mindful of the possibility of impaired absorption of oral desmopressin during acute gastrointestinal dysmotility, particularly in patients with adipsic AVP-D who lack an intact thirst mechanism.

## Learning points

- Patients with adipsic AVP-D lack the protective thirst mechanism that buffers errors in fluid or desmopressin dosing, making them highly susceptible to clinically significant fluctuations in serum sodium.
- Severe hyponatremia and its correction may represent an under-recognized precipitant of ACPO and should be considered in the differential diagnosis of new-onset colonic dilation during sodium correction.
- Because oral desmopressin has low bioavailability, acute gastrointestinal dysmotility may further impair its absorption. Concurrent PEG 3350 administration may be an additional contributing factor, although this association requires further investigation.

## Data Availability

Data sharing is not applicable to this article as no datasets were generated or analyzed during the current study.
